# Development of a Comprehensive Database System for Safety Analyst

**DOI:** 10.1155/2015/636841

**Published:** 2015-06-08

**Authors:** Alexander Paz, Naveen Veeramisti, Indira Khanal, Justin Baker, Hanns de la Fuente-Mella

**Affiliations:** ^1^Department of Civil and Environmental Engineering, Transportation Research Center, 4505 Maryland Parkway, P.O. Box 454015, Las Vegas, NV 89154-4015, USA; ^2^Transportation Research Center, College of Engineering, University of Nevada, Las Vegas, 4505 S. Maryland Parkway, P.O. Box 454015, Las Vegas, NV 89154, USA; ^3^Facultad de Ciencias Económicas y Administrativas, Escuela de Comercio, Pontificia Universidad Católica de Valparaíso, Avenida Brasil 2830, 2340031 Valparaíso, Chile

## Abstract

This study addressed barriers associated with the use of Safety Analyst, a state-of-the-art tool that has been developed to assist during the entire Traffic Safety Management process but that is not widely used due to a number of challenges as described in this paper. As part of this study, a comprehensive database system and tools to provide data to multiple traffic safety applications, with a focus on Safety Analyst, were developed. A number of data management tools were developed to extract, collect, transform, integrate, and load the data. The system includes consistency-checking capabilities to ensure the adequate insertion and update of data into the database. This system focused on data from roadways, ramps, intersections, and traffic characteristics for Safety Analyst. To test the proposed system and tools, data from Clark County, which is the largest county in Nevada and includes the cities of Las Vegas, Henderson, Boulder City, and North Las Vegas, was used. The database and Safety Analyst together help identify the sites with the potential for safety improvements. Specifically, this study examined the results from two case studies. The first case study, which identified sites having a potential for safety improvements with respect to fatal and all injury crashes, included all roadway elements and used default and calibrated Safety Performance Functions (SPFs). The second case study identified sites having a potential for safety improvements with respect to fatal and all injury crashes, specifically regarding intersections; it used default and calibrated SPFs as well. Conclusions were developed for the calibration of safety performance functions and the classification of site subtypes. Guidelines were provided about the selection of a particular network screening type or performance measure for network screening.

## 1. Introduction

The Highway Traffic Safety Grants from the National Highway Traffic Safety Administration (NHTSA) for Fiscal Year (FY) 2013 were estimated to be $643 million [1]. Motor vehicle crashes are of critical concern in the United States and an enormous amount of resources is spent on highway traffic safety. Based on statistical projections from NHTSA's Fatality Analysis Reporting System (FARS), traffic fatalities increased from 32,367 in 2011 to 34,080 in 2012, a 5.3% increase. In fact, 2012 was the first year since 2005 to have a year-to-year increase in fatalities, which indicates that considerable work is needed to improve highway safety [2].

FHWA's Highway Safety Improvement Program (HSIP) is a critical component of the safety provisions in the Moving Ahead for Progress in the 21st Century Act (MAP–21, P.L. 112–141) [3]. As a part of HSIP, state Departments of Transportation (DOTs) have developed a Strategic Highway Safety Plan (SHSP) to identify, analyze, and address traffic safety problems. State-of-the-art tools have been created to support the development of SHSP and generate better traffic solutions for existing and emerging safety problems. Some of these tools include the Interactive Highway Safety Design Model (IHSDM), the* Highway Safety Manual* (HSM), and the software tool, Safety Analyst. These tools can be used by DOTs to satisfy MAP-21's performance-based federal program, which mandates that state DOTs must establish safety performance targets and achieve them within two years [3]. This requires a program for highway safety management that should include

- identifying locations with potential for safety improvements,
- diagnosing identified locations for safety improvement and countermeasure selections,
- estimating the cost of the countermeasures,
- estimating the benefits of the countermeasures.

These new tools address many limitations of traditional safety analysis tools, including bias associated with volume, segment length, and regression-to-the-mean as well as incorrect model forms and the lack of reliability measures [4–9]. In order to address these limitations, state-of-the-art tools, including Safety Analyst, use analytical methods that require comprehensive datasets in order to provide sufficient information and capture intricate spatiotemporal characteristics and interactions in the traffic system.

In their FY2013 budget estimate, NHTSA had determined that to reduce highway injuries and fatalities, highway safety programs that are data-driven and self-sustaining needed to be developed and implemented [1]. The federal government has allocated considerable resources to build accurate and timely safety datasets at the national and state levels [10]. Key safety data has included information about crashes, roadways, traffic flow, driver history, citation/adjudication, and construction projects [4, 11]. This data has been required by a number of other safety programs, such as the Highway Rail Grade Crossing Program and the High Risk Rural Road Program. Currently, various divisions at many state DOTs collect and maintain datasets based on their data needs, and some of this data is shared across divisions. However, this approach may not be the best for a number of following reasons:

- Not all interested groups are aware of the availability of data at each division.
- There is no consistency in terms of how the information is stored and the data normalized.
- Typically, the datasets are developed without explicitly considering the needs of the various applications used by different divisions.
- New emerging tools, such as Safety Analyst, require data to be collected from multiple divisions. In addition, these tools need data that typically are not available.
- The training of traffic safety engineers and professionals on the use of new applications, such as Safety Analyst, requires the corresponding applications to be ready for use with all the necessary data available.
- Coordination with other statewide public safety agencies requires a comprehensive approach to integrate and enable access to the data as well as to provide maintenance capabilities.

A comprehensive approach using state-of-the-art tools is required to collect data and manage existing data needs, which are significant, as well as to develop better solutions. The existing literature is populated with examples about data collection and integration methods for transportation applications, including frameworks for geographic information systems (GIS) [12–18], database/data-warehouse systems [19–24], and visualization tools [25–27]. However, most DOTs do not have access to a comprehensive database system that enables them to take full advantage of existing tools, including Safety Analyst. With such a database system, agencies may be able to develop safety performance functions (SPFs) that are jurisdiction-specific so that they could estimate performance measures more accurately. Previous studies show that methodologies to develop such systems are relatively limited.

Many state DOTs have large amounts of data. However, it is a herculean task for them to identify the data sources, develop the systems to integrate them, and develop the databases, analysis tools, and visualization systems. This study has created and tested a database system and tools to process, integrate, check, and load the data so as to provide data to multiple transportation applications with the focus on Safety Analyst. The tools developed in this study can be used to create similar database systems for any region and/or to expand existing databases. A recent nationwide survey [28] has revealed that a major deterrent for using Safety Analyst is the unavailability of comprehensive data sources as well as tedious methods for data importing and processing. Hence, considering the substantial resources invested in developing Safety Analyst as well as the significant potential advantage of conducting Traffic Safety Management using such tool, the contributions of this study are timely and can facilitate to increase the use and benefits of Safety Analyst.

**Figure 1:**
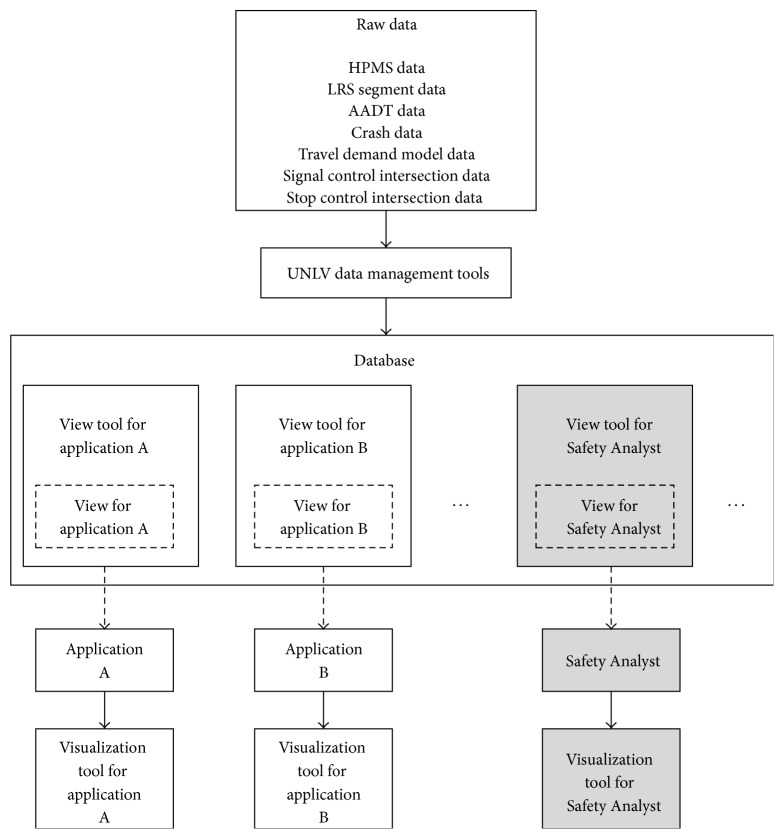
Conceptual framework for the comprehensive database and visualization system developed in this study.

Figure 1 illustrates the conceptual framework for the proposed database system and a companion visualization system with corresponding work was published elsewhere [29]. Raw data was processed using data management tools to create a comprehensive, normalized, and optimized database. View tools are used to provide the data required by each application in the corresponding format and level of resolution. Visualization tools are used to provide multiple graphical representations of the inputs and outputs for each application. Many analysis tools that exist currently, including Safety Analyst, do not provide visualization capabilities. Considering the spatial nature of the problem, this was a significant limitation.

## 2. Safety Analyst

Safety Analyst provides a suite of analytical tools to identify and manage system-wide safety improvements [5]. Safety Analyst uses an empirical Bayesian (EB) method as an alternative to traditional safety analysis methods, such as frequency, rate, critical rate, or crash index. The EB approach provides a mechanism that cannot be addressed using traditional methods and which addresses issues associated with bias, incorrect model form, and the lack of a reliability measure [4–10].

Safety Analyst consists of four tools: administration, data management, analysis, and countermeasure implementation. The administration tool includes federal, agency, and system components [30]. The federal component provides access to the default site subtype definitions, countermeasure management, and national default Safety Performance Functions (SPFs). The agency component provides access to various operations, including adding, changing, and removing data attributes, with the exception of mandatory data attributes. Further, this component enables the modification of national SPFs with agency-specific SPFs. The system component maintains local or remote databases and combines the database with federal and agency components.

Local or remote databases can be imported using the data management tool [30]. Currently, Safety Analyst supports two basic mechanisms for data import: a file import and database-to-database mapping. For DOTs that maintain a complete data inventory in a database management system (DBMS) that is compliant with structured query language (SQL), the database-to-database mapping mechanism is the best alternative with which to load data into Safety Analyst. The view tools developed in this study provide this feature. For DOTs that do not maintain a database with all the required data for Safety Analyst, the data management tools developed in this study can be used to generate a DBMS with all the required data.

The file import is a less desirable mechanism because it does not provide all the capabilities of having the data in a DBMS. Safety Analyst supports data inventory files in extensive formats for mark-up language (xml) and comma-separated value (csv). However, the inventory files have to satisfy a particular format. It is unlikely that DOTs have readily available xml or csv datasets that satisfy the required format. Hence, developing a DBMS for Safety Analyst is recommended.

The analysis tool used to perform various analyses [30] has a set of four modules, including the following [4, 30]:

- *Network Screening Module*: this module identifies and ranks sites using the EB method for potential safety improvements.
- *Diagnosis and Countermeasure Selection Module*: this helps to diagnose safety problems at specific sites, using answers provided by the user for a set of built-in questions. Based on the diagnosis, the user can select countermeasures to reduce crash frequency and severity at specific sites.
- *Economic Appraisal and Priority Ranking Module*: this provides an economic evaluation of a specific countermeasure for a specific site or several alternative countermeasures for multiple sites. Further, it provides priority ranking of sites and proposed improvement projects based on benefit and cost estimates.
- *Implemented Countermeasure Module*: this module provides before/after evaluations of implemented safety improvements. Data for construction projects and implemented countermeasures are required. This data can be imported using the countermeasure implementation tool.

### 2.1. Data

**Figure 2:**
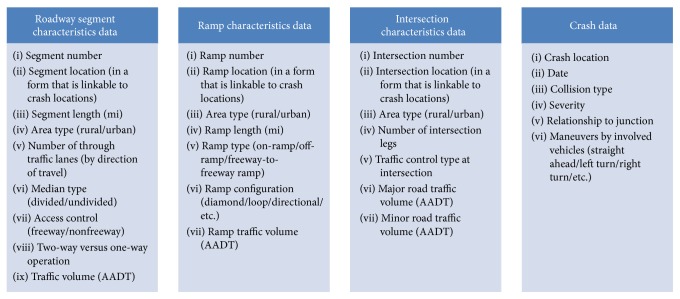
Mandatory data elements required by Safety Analyst.

Critical data to perform traffic safety studies include crash, roadway, control, and traffic flow. A comprehensive plan for data collection was developed to obtain available data from various state agencies in Nevada, based on the Model Minimum Uniform Crash Criteria (MMUCC) and the Model Inventory of Roadway Elements (MIRE) [31, 32]. Based on these guidelines, approximately 150 data attributes were necessary for the development of a comprehensive safety database. Not all the data was required by Safety Analyst; however, in this study, a data dictionary was developed to explicitly identify the mandatory data for Safety Analyst [4, 30], as shown in Figure 2.

Most of the data in Figure 2 is available from various DOT sources, including FHWA's Highway Performance Monitoring System (HPMS); linear referencing systems (LRS) of road networks; travel demand models (TDM); and intersection, traffic volume, and crash datasets [26]. For this study, data for roadway segments and ramps were obtained from the LRS, HPMS, and TDMs. Crash data were obtained from the Nevada Accident and Citation Tracking System (NCATS). Annual average daily traffic (AADT) was collected from NDOT's Traffic Records Information Access (TRINA).

### 2.2. Road Network

A road network is the centerline map of routes in a GIS LRS. Most of the state DOTs have two levels of road networks, a state-level dataset (SDS) and a county-level dataset (CDS). The SDS can be used for federal aid and national highway system roads in Safety Analyst, and the CDS can be used for county-level minor arterial roads as well as for major and minor collector roads. Typically, an SDS road network is similar to an HPMS routes layer. When both SDS and CDS road networks are unavailable, the HPMS routes layer in LRS [33] can be used with some modifications.

For this study, the CDS road network in LRS was used, which included an additional system, RouteMaster identification (RMID), a unique identifier for referencing the route in the road network. RMID improves the ability to reference other data sources to the road network. Road network data includes the segment ID, RMID, the type of road, the county, the beginning and end mileposts of the segment, the cardinal direction (the direction in which the road begins and ends), and the length of the segment.

### 2.3. HPMS Data

The Highway Performance Monitoring System is a national-level system maintained by the FHWA and includes data on the extent, condition, performance, use, and operating characteristics of state-owned and some non-state-owned highways [33]. The HPMS data model by the FHWA, which is in a GIS framework, provides the spatial relationships among the data elements. FHWA mandates the state DOTs to submit complete timely and accurate HPMS data every year [33]. Hence, this data, integrated with other data sources, can be available to state DOTs for database development required for Safety Analyst.

For this study, Nevada HPMS data layers were used, including access control, facility type, functional classification, speed limit, through lanes, AADT, and urban code.

### 2.4. Travel Demand Model

Usually, urban metropolitan planning organizations (MPOs) have a GIS-based TDM for transportation planning and transportation improvement programs. The data from this model, such as number of lanes, speed limit, access control, functional classification, area code, travel direction, one-way or two-way, and ramp configuration, can be used when HPMS data is not available. If a distinct county-level road network is not available, a TDM road network can be used for data on road segments, ramp segments, lengths, and mileposts.

For this study, the TDM of the Regional Transportation Commission of Southern Nevada (RTC-SN) was used to obtain data not available in the HPMS layers.

### 2.5. Crash Data

Every year, NHTSA spends much of its budget on their highway safety grants for the Crash Data Collection Program [1]. The collection of crash data from states must be based on MMUCC guidelines. The crash data required by Safety Analyst is based on MMUCC guidelines as well. This study used data from NCATS for located crashes (crashes with coordinates) and crash characteristics for the years 2007 to 2011.

### 2.6. AADT Data

Safety Analyst requires AADT for all the segments to be used in a network-level analysis. Frequently, however, these data are not available for all roadway classes. Typically, state DOTs collect data to estimate AADTs for high functional classes of roads, such as freeways and state roads. Collecting similar data for arterials and local roads is an extensive and expensive process. This study used a simulation-based dynamic traffic assignment model, DynusT [34, 35], to estimate the AADT for those locations with missing AADT for the latest year. These AADTs were projected for five years by using temporal factors developed from long-term counts.

### 2.7. Intersection Data

Typically, county agencies or metropolitan planning organizations (MPOs) have data for signalized intersections, including the location and type of control information. However, data for stop-controlled intersections is not common and needs to be collected. In this study, a methodology and a tool were developed to collect stop-control data efficiently. Signalized intersection data was obtained from the Freeway and Arterial System of Transportation (FAST), a division of RTC-SN.

Table 1 shows the source files typically available in state DOTs and/or MPOs as well as data in those files that are required by Safety Analyst. With this information, agencies can start collecting these files to develop a Safety Analyst database. Agencies can choose either (1) HPMS files having data for road networks and crashes for roads maintained by state DOTs or (2) HPMS files with data for road networks, TDMs, crashes, and intersections for county-level roads.

The road networks, along with HPMS files, including AADT and crash data, form an integrated database covering all state-owned roadways and ramp segments, at least. However, this study required data on county-level roads as well. Therefore, data from the CDS road network was integrated with data from HPMS layers, TDM, intersections, AADT, and crashes. During integration, some of the issues found among these datasets are as follows:

- A spatial shift/gap exists among GIS shape files of various datasets, such as HPMS, CDS road network, and TDM layers.
- No common ID are present among HPMS, CDS road network, and TDM layers.
- Segmentation lengths differ in HPMS layers and the CDS road network.
- There is no unique RMID among the datasets.
- Some data are incorrectly represented, such as ramp configurations and the number of lanes.

Certain issues in the datasets are common because there is no consistency in data formatting and storage among divisions or departments. Furthermore, the collected data may or may not have been stored in the same geographical format, such as cardinal measurements, coordinate systems, and geometry. ArcGIS ModelBuilder [36] was used to develop the automated tools to solve these issues, as will be discussed in the following section.

## 3. Data Management Tools

### 3.1. Data Collection Tool

Even though multiple data sources exist that provide a vast amount of the required data for Safety Analyst, various data attributes were missing or incomplete, including ramp type, ramp configuration, and the type of control at intersections. Most of the missing data were collected using Google Earth, and the missing information was observed and coded in Google Earth as well. A data collection tool was developed to extract data as well as create ArcGIS shape files with all the information. This capability facilitated the development and integration of the database.

Safety Analyst required that all collected data be integrated using (1) a route and a milepost; (2) a route, county, and milepost; (3) a route, section, and distance; or (4) a section and distance. This study used a route and milepost index to integrate all the data because some of the datasets had this information. Although various commercial methods and tools are available [12–18] to integrate the data, integration tools using ArcGIS ModelBuilder were developed in this study to gain total control of the process and provide greater automation.

### 3.2. ArcGIS ModelBuilder Tool

ModelBuilder [36] is an application existing inside ArcGIS by which models can be created, edited, and managed. A model is built with a sequence of processes and data chained together. Once built, a model can be saved as a tool and embedded in an ArcMap toolbar. The two primary uses of ModelBuilder are to execute a process sequence that was created and to create additional tools with new capabilities. These tools can be launched from the tool dialog box or from Python scripts. Using the ModelBuilder tool, the following operations can be performed:

- Change parameter values, such as buffer radius or tolerance limits, and rerun the models.
- Add more processes, such as components for a buffer or intersect, as well as data.
- Delete processes and intermediate data.
- Visualize and explore the results in ArcMap.

ModelBuilder tools were developed to overcome all the data issues encountered with HPMS, road networks, TDM, and AADT. The three primary tools used were

- a mapping tool that maps road network segments spatially to data elements in HPMS, when there is geometry shift and no common field between them;
- a linear referencing tool that creates a milepost index for each crash with respect to roadway segments, ramps, or intersection mileposts;
- a dynamic segmentation tool that breaks/joins the segments at required locations.

### 3.3. Interface for Data Attribute Mapping

An interface for data-attribute mapping was developed to populate the database, using data from existing sources. The interface established mapping for every attribute as well as data sources from user-file data attributes to corresponding database attributes in the database tables. This interface enabled using existing data files without any modifications. The interface used a Microsoft Excel spreadsheet (.xlsx), which is a metadata file with four columns. The first and second columns included the database table name and the attributes name, respectively. These names were fixed and did not need to be changed. The third and fourth columns included the user's (agency) file name and attribute name, respectively.

Table 2 illustrates the metadata file. Only the user file name and user file attribute name have to be filled out by the user. Different DOTs store their data in various files, and common unique ID relate those files and attributes. For example, Nevada has roadway attributes in such files as CDS_Network, Las Vegas Median, HPMS_Access, and HPMS_SpeedLimit. Once a metadata file is filled in, the data-attribute mapping interface is used to insert and store data from the user file into the corresponding database tables and attributes.

### 3.4. Data Instantiation and Insertion

Inputs to an existing database can be either new or an update of previously inserted data. A data instantiation and insertion tool was developed to input data into the database, taking into consideration the interdependencies of the data. Input files were streamed and parsed with a simple application-programming interface (API) for an XML parser, also known as SAX, which was used to store the data in a matrix.

When a row is read in the matrix, a “select” query is performed on the database to determine the existence of an object. If an object exists, an update is performed: a Java object is instantiated, and its fields are updated with the values in the input file. Then, the update method of this Java object is processed to update the database. If there is no matching object, a new object is instantiated and inserted into an “EntityManager” class. Once all files are parsed and all the objects instantiated, the data can be inserted into the database.

The “EntityManager” class handles the priorities of the tables automatically in order to satisfy interdependencies between tables. Inputting data without using EntityManager might lead to either data insertion failure or database corruption due to a violation of table interdependencies. For example, accident vehicle data is dependent on accident data, and a mechanism is required to account for this dependency.

## 4. Database Schema

The database schema provides the structure of a DBMS, which is described in a formal modeling language. Current database-modeling languages include the entity-relationship (ER) model and the unified modeling language (UML). ER is a conceptual data model that views the real world as entities and relationships. The basic constructs in an ER model are the entities, attributes, and relationships that are in an ER diagram. The ER model focuses on the conceptual and logical design phase of the database. It can be used to develop SQL-compliant database systems, which are convenient for users unfamiliar with database operations [37].

The UML is an object-oriented visual modeling language used to specify, visualize, analyze, and control the objects of a software system. It is used to understand, design, browse, configure, maintain, and control information about software systems [37]. This study used the ER model for three important reasons. First, Safety Analyst only supports SQL-compliant databases. Second, ER diagrams, which reveal the design of the database, are easier to understand compared to UML diagrams. Third, most applications similar to Safety Analyst are more likely to be compatible with an ER model.

The physical data model for the database was built using the ER model, which indicates how data should be represented and stored by a DBMS, such as Oracle, MySQL, SQLServer, or Derby [38]. In this study, the user had the option to choose either MySQL or Derby as a comprehensive database system. However, for the Safety Analyst View, only Derby was enabled because MySQL is not compatible with Safety Analyst. Both databases are open-source, SQL-compliant DBMSs and provide all the required capabilities of a reliable, flexible, and robust DBMS. SQL scripts were developed to generate database tables and the relationships among them in MySQL and Derby.

The physical data model for the database was created, and then the database was populated with data. Data insertion is a process that can happen once, periodically, or sporadically. The methodology to populate the database was designed to account for most potential scenarios that could arise. For example, various empty tables were designed and created for future data that may become available and/or desirable.

**Figure 3:**
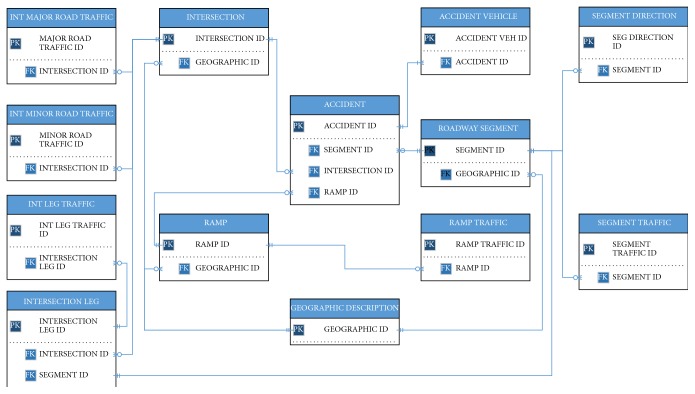
Entity relationship diagram for the proposed safety database.

**Figure 4:**
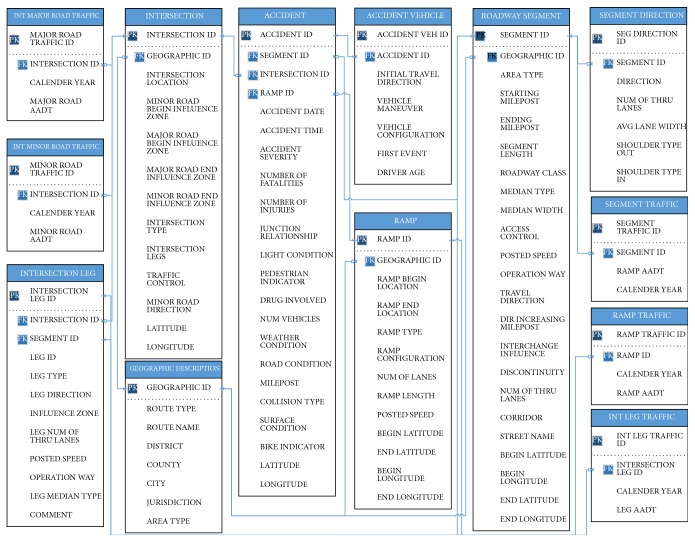
A physical data model for the safety database.

A complete entity relation diagram, including tables, primary key, and foreign keys, of the database is provided in Figure 3. This study has used Crow's Foot Notation to illustrate entities and relationships in the proposed database. An entity is a data storage container (table) with a collection of attributes. In Figure 3, ACCIDENT is one of the entities. It has a primary key identifier marked with PK, which uniquely identifies one instance of an entity and a foreign key identifier marked with FK, which uniquely identifies a row of other entity. A relationship illustrates an association between two entities and it consists of indicators of the business rules. In Figure 3, the relationship between the entities in the database is illustrated using the blue lines and symbols for beginning and ending indicators of the business rules. The symbols used are two vertical lines for “one and only one” indicator, circle with crow feet for “zero or many,” one vertical line with crow feet for “one or many,” and circle with a vertical line for “zero or one indicator.” For example, in Figure 3, zero or many “SEGMENT ID” from “ACCIDENT” entity are associated with the one and only “SEGMENT ID” from “ROADWAY SEGMENT” entity. For more details about ER diagram, readers can refer to data modeling with ER diagrams by Riccardi [39]. In addition, Figure 4 illustrates a detailed data framework using physical data model including tables, primary and foreign keys, and other attributes for the proposed safety database.

Such analysis tools as Safety Analyst require data in a particular format. For example, Safety Analyst requires crash severity type in the form of “K” for fatal, “A” for severe injury, and “P” for property damage. However, it is unlikely that the data sources use the same formatting. Having the requirement to follow a particular formatting is one of the primary barriers for DOTs to use Safety Analyst [28]. The database developed in this study stores the type of crash severity in the form of fatal, injury, or property damage.

A view tool for Safety Analyst was developed to provide a database view consistent with the requirements of Safety Analyst.  Table 3 illustrates a portion of an MS Excel sheet used to establish mapping between the general database view and the Safety Analyst view. Database Table Name, Attribute Name, and Attribute Values were mapped between the two views. For example, in Table 3, the Database Table Name is “accident,” the Attribute Name is “severity,” and Attribute Values are “fatal injury,” “severe injury,” and “property damage only.” The corresponding Safety Analyst values are Accident; accidentSeverity1; and K, A, or P. The back-end of the view tool for Safety Analyst has an MS Excel parser that streams the data, provides mapping, and stores the data in a matrix. HashMaps are created and a relationship is established between the database and the Safety Analyst view.

## 5. Analysis and Results

The comprehensive database as well as the database view of Safety Analyst for Clark County, Nevada, was developed with the proposed data management tools and populated using the data sources described earlier. Using the data management tools, the database view was mapped, imported, and postprocessed. Calibration factors for various site subtypes were (1) urban freeway segments with four and six lanes; (2) urban freeway segments in interchange areas with four and six lanes; (3) urban signalized four-leg and three-leg intersections; (4) urban stop-controlled with four-leg and three-leg intersections; and (5) arterial segments with two, four, and six lanes. These factors were obtained by calibrating the federal default SPFs, using Nevada data. Network screening analysis was performed using the analytical tool in Safety Analyst to determine sites with the most potential for safety improvements.

Network screening analysis can be performed using multiple combinations of screening types, safety performance measures, severity, and screening attributes. Results can be reported using two types of reports: (1) conventional, with all the site results and (2) a percentage type, specifying the percent (e.g., the top 5% sites). Three basic screening types are available that can report

- the expected and excess crash frequencies, with peak searching on roadway segments using limits for the coefficient of variation (CV) [8, 30, 40];
- a sliding window on roadway segments;
- corridor screening [8, 30, 40].

Other screening types analyze a high proportion of specific crash types, a sudden and steady increase in mean frequency, and corridor screening [30, 40].

Safety performance measures, expected and excess crash frequencies for different severities, and various screening attributes can be computed [8, 30, 40]. Using the database for Clark County, various analyses using different network screening methods with default SPF (calibration factor = 1) and calibrated SPF were conducted for

- analysis of roadway and ramp segments and intersections;
- analysis of roadway segments based on functional classifications;
- analysis of signalized and stop-controlled intersections;
- analysis of ramp segments.

To illustrate the results, this paper examines two case studies that used excess crash frequency as a safety performance measure to see if crashes were reduced if a safety improvement was implemented [30]. The first case study identified the top 5% sites, including roadway and ramp segments as well as signalized and stop-controlled intersections, which have the potential for safety improvements.

Two analyses, with default and calibrated SPFs, were performed. Excess crash frequency was calculated for fatal and all injury crashes, with peak searching on roadway segments having coefficient-of-variation (CV) limits for the entire network. The peak-searching screening type was used because it had CV-limit statistics and a minimum window length of a 0.1-mi segment. Hence, the exact section/window of the site that had the potential for safety improvement could be determined to deploy a countermeasure. Seven out of 10 sites proved to differ in the top ranks.  Table 4 shows the results of the first case study, including the top 10 sites (the first 10 ranks) having the potential for safety improvements. These sites consisted of two site subtypes, four-lane segments of an urban freeway in the interchange area (Site Subtype 158) and multilane divided segments of an urban arterial (Site Subtype 153). Site Subtype 158 had a lower calibration factor, 0.17, implying that these roadways experienced fewer crashes, on average, than roadways used to develop the federal SPFs for Safety Analyst. Conversely, Site Subtype 153 had a higher calibration factor, 4.27, implying that these roadways experienced higher crashes, on average, than roadways used to develop federal SPFs. Hence, yearly calibration of SPFs plays a significant role in screening sites that have a higher potential for safety improvements.

**Figure 5:**
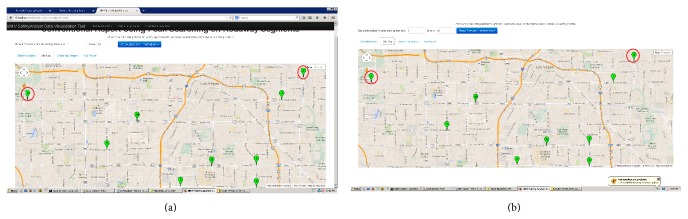
(a) Results of a basic network screening for fatal and all injury crashes at intersections, using default SPF, and (b) results of basic network screening for fatal and all injury crashes at intersections, using calibrated SPFs.

Using default and calibrated SPFs, the second case study identified intersection sites with the potential for improvements regarding both fatal and all injuries. The excess crash frequency for fatal and all injury crashes was calculated. Figures 5(a) and 5(b) illustrate top 10 intersection sites (the first 10 ranks) having a potential for safety improvements. When analyzing default and calibrated SPFs, two sites (circled in red) interchanged Ranks 4 and 5; this was because the sites with Rank 3 and 4 had different site subtypes; hence, different calibration factors were used.

The top 10 sites consisted of two different site subtypes, the urban four-leg signalized intersection (Site Subtype 253) and the urban three-leg intersection (Site Subtype 254). Site Subtype 253 had a slightly higher calibration factor, 1.08, and experienced more crashes than the intersections used to develop federal SPFs. Site Subtype 254 had a lower calibration factor, 0.64, implying that urban three-leg signalized intersections experienced less crashes than the intersections used for developing federal SPFs of such sites.

In the results, the predicted crash frequency was much less when compared to the observed crash frequency due to the default SPF in Safety Analyst. The predicted crash frequency was one of the important measures when calculating the expected or excess crash frequency by the EB method. Because of the urban nature of the study area, higher levels of AADT (100,000 s) worsened these results.

Currently, Safety Analyst calibrates the default coefficients estimated based on national-level data for various site subtypes, such as two-lane freeways, four-lane freeways, by using agency AADT data. This issue can be solved in two ways:

- Create agency-specified site subtypes with different AADT ranges in the administration tool, and recalibrate the coefficients.
- Based on the data, develop separate count-regression models for site subtypes, and input the coefficients in the administration tool.

Because there are minimum guidelines about a screening type or performance measure to choose for specific analysis, in this study, many case studies were tested to infer the Safety Analyst results. From the inference of results, the following conclusions were obtained:

- Peak searching screening type was not a good parameter for segments less than 0.1 mi. It proportionated expected/excess crash frequencies for 0.1 mi when the length was less than 0.1 mi. In this case, a sliding window was a better choice because it aggregated and moved the window on contiguous segments for a calculation; further, it proportionated expected/excess crash frequencies for 0.3 mi, the minimum length used to calculate performance measure, when the length of site was less than the window length.
- In Safety Analyst, peak searching was better because it had the coefficient-of-variation limit, whereas the sliding window did not.
- Peak searching was not a good parameter for longer segments. Peak searching provided one rank per site, with a window length of 0.1 mi. Other windows having the second highest expected/excess crash frequencies were provided in additional windows, with longer segments having multiple additional windows. However, the sliding window provided consecutive ranks for the same site with various windows.
- For sites with a higher number of crashes and a large variance, analysts could use either expected or excess crash frequencies.
- No particular screening type was preferred for the entire analysis. Analysts are encouraged to analyze a given site list using multiple combinations of network screening to find common sites from the output. When the same site is identified using several screening methods, this reinforces that the site deserves further investigation [30].

## 6. Visualization Tool for Safety Analyst

The output capabilities provided by Safety Analyst are limited to tables that report the results in HTML, PDF, RTF, and CSV formats. Analysts have to infer the results from these voluminous tables without having an image of the site. Hence, a visualization tool was developed [29] to provide better meaning to the output, with expanded capabilities for spatial, graphical, and editable reports. To visualize the results using the visualization tool, the user can choose between two alternative display methods: Google Maps and ArcGIS. The advantage of using Google Maps is its simplicity and availability; the advantage of ArcGIS is its modeling and computing capabilities.

For the Google Maps interface, the visualization tool has a web-based front end; for the ArcGIS interface, the visualization tool is a standalone application based on ArcPy scripts. Both applications provide easy access to multiple tabs. The first two tabs display the tabular results and a map with spatial locations. In addition, the user can interact with the graphical display to perform such basic operations as zoom in, zoom out, and select sites. In the second tab, the user can choose the bar chart for the safety performance measures, such as observed, predicted, and expected/excess crash frequencies for several sites.

Interpreting the results by means of graphs is easier than by using tables. The user can include site spatial locations and performance measure bar charts in the editable Safety Analyst report in the third tab. The tabular results of network screening provided in Table 4 are difficult to use without the visualization tool. However, Figures 5(a) and 5(b) illustrate the spatial locations of intersections by using the visualization tool [29], determined by means of network screening.

## 7. Conclusions

The benefits of developing and using a comprehensive database system for traffic safety studies are significant. This study developed a comprehensive database system that can provide data to multiple applications for traffic safety engineering and other potential needs. Furthermore, it provided the methodology and guidance to develop a database from the existing, readily available data sources of state DOTs and/or MPOs. The tools developed to build the comprehensive database and view for Safety Analyst can be used by other agencies, as they use noncommercial software. This system allows the use of state-of-the-art traffic safety tools to support the development of federal requirements as well as to develop better traffic safety solutions for existing and emerging problems. These tools offer significant savings in terms of time, money, and lives.

In particular, the proposed database system has the capability to provide data to Safety Analyst, the state-of-the-art highway safety management software. Although Safety Analyst provides tremendous analysis capabilities, few agencies take advantage of these capabilities because the software requires significant data needs, complex development of the required inputs, and lack of experience and knowledge in creating the inputs as well as using the software [26]. The proposed database system, along with its data management and visualization tools, provides significant support to circumvent these barriers. This database system can be used to develop jurisdiction-specific SPFs tor estimate of performance measures more accurately and precisely.

This study could be expanded to develop tools that create different site subtypes based on the data in the Safety Analyst view. SPFs could be developed for those site subtypes, and the coefficients could be inputted into the administration tool to obtain better predictions for crash frequency. Predictive methods in Part C of the* Highway Safety Manual* [10] could be used to develop SPFs. In this case, the developers of Safety Analyst should expand the capabilities of an administration tool to accommodate the agency-specific multiparameter SPFs and their coefficients.

## Figures and Tables

**Table 1:** Source files and their data elements to build a safety database.

Road network	HPMS files	TDM model	Crash data	Intersection
Segment ID	Routes	Travel direction	Accident ID	Intersection ID
Ramp ID	Functional classification	Functional classification	Crash location	Intersection location
Segment length	Access control	Operation way (one- or two-way)	Cash date	Type of control
Begin milepost	Speed limit	Speed limit	Collision type	Number of legs
End milepost	Through lanes	Number of lanes	Severity	
Route ID	Lanes_Left	County	Relationship to junction	
County	Lanes_Right	Area code	Direction of involved vehicle	
Increasing milepost direction	AADT	Ramp configuration (sometimes available)	Maneuvers by involved vehicle	
	Urban	Ramp type (sometimes available)		
	County	Segment ID		
		Ramp ID		
		Segment length		

**Table 2:** Sample of the metadata file for data mapping.

Database table name (fixed)	Database attribute name (fixed)	User file name (to be filled by user)	User file attribute name (to be filled by user)
RoadwaySegment	agencySegmentID	CDS_Network	ID1
RoadwaySegment	beginLocation	CDS_Network	Beg_Route_
RoadwaySegment	endLocation	CDS_Network	End_Route_
RoadwaySegment	routeName	CDS_Network	Route_MAST
RoadwaySegment	routeType	CDS_Network	Route
RoadwaySegment	county	CDS_Network	County_code
RoadwaySegment	length	CDS_Network	Datum_Seg2
RoadwaySegment	terrain	HPMS_terrain	Terrain
RoadwaySegment	roadwayClass	CDS_Network	F08_FType1
RoadwaySegment	medianType	LasVegas_Median	MedianType
RoadwaySegment	accessControl	HPMS_Access	Value_name
RoadwaySegment	medianWidth	LasVegas_Median	MedianWidth
RoadwaySegment	postedSpeed	HPMS_SpeedLimit	PostedSpeed

**Table 3:** Mapping between a general and the safety analyst views.

Database table name	Attribute name	Attribute values	Safety analyst view table name	Safety analyst view attribute/name	Safety analyst view attribute value
Accident	Severity	Fatal injury	Accident	accidentSeverity1	K
Accident	Severity	Severe injury	Accident	accidentSeverity1	A
Accident	Severity	Property damage only	Accident	accidentSeverity1	P
RoadwaySegment	routeType	State route	RoadwaySegment	routeType	SR
RoadwaySegment	routeType	Interstate	RoadwaySegment	routeType	I
RoadwaySegment	routeType	U.S. route	RoadwaySegment	routeType	US
RoadwaySegment	roadwayClass	Principal arterial, other	RoadwaySegment	routeType	3
RoadwaySegment	roadwayClass	Minor arterial	RoadwaySegment	roadwayClass	4
RoadwaySegment	roadwayClass	Local	RoadwaySegment	roadwayClass	7
RoadwaySegment	roadwayClass	Major collector	RoadwaySegment	roadwayClass	5
RoadwaySegment	roadwayClass	Principal arterial, other freeways or expressways	RoadwaySegment	roadwayClass	2
RoadwaySegment	roadwayClass	Principal arterial, interstate	RoadwaySegment	roadwayClass	1
RoadwaySegment	roadwayClass	Minor collector	RoadwaySegment	roadwayClass	6
RoadwaySegment	roadwayClass	Other	RoadwaySegment	roadwayClass	0
RoadwaySegment	roadwayClass	Unknown	RoadwaySegment	roadwayClass	99

**Table 4:** Results of basic network screening with peak searching on roadway segments and CV tests from safety analyst for fatal and all injury crashes on roadway and ramp segments as well as intersections, using default, and calibrated SPFs.

Analyses type	Rank	Site Subtype	Route	Location with highest potential for safety improvement							
				Start Location	End location	Average observed crashes^*∗*^	Predicted crash frequency^*∗*^	Excess crash frequency			
								Excess frequency^*∗*^	Variance^*∗∗*^	Number of fatalities	Number of injuries
Default SPF calibration factor (CF) = 1.0	1	158	IR15	40.223	40.323	267.05	21.49	219.41	408.32	2.04	312.21
	2	153	SR589	3.311	3.411	154.14	4.63	139.86	149.66	1.51	211.41
	3	158	IR15	41.386	41.486	173.08	27.10	133.70	609.28	1.24	190.24
	4	158	IR15	35.112	35.768	142.14	23.27	107.04	451.76	0.99	152.32
	5	153	SR612	4.605	5.124	114.01	5.28	102.18	182.06	1.10	154.46
	6	153	SR593	0.889	1.574	103.06	9.51	90.25	544.53	0.97	136.42
	7	153	SR159	29.664	30.193	101.96	8.06	90.19	396.04	0.97	136.33
	8	153	SR612	5.124	5.633	98.84	2.63	86.57	53.97	0.93	130.86
	9	153	SR593	3.784	6.361	94.92	3.99	84.30	107.53	0.91	127.42
	10	153	Las Vegas Blvd.	26.032	26.112	87.10	6.22	75.67	239.96	0.82	114.37

Calibrated SPF Site Subtype 158 CF = 0.17 Site Subtype 153 CF = 4.27	1	158	IR15	40.223	40.323	291.56	20.43	238.49	379.89	2.21	339.36
	2	158	IR15	41.386	41.486	189.06	25.76	147.70	558.06	1.37	210.18
	3	153	SR589	3.311	3.411	156.48	19.18	135.02	2165.74	1.46	204.09
	4	158	IR15	35.668	35.768	155.78	22.12	118.56	414.43	1.10	168.71
	5	153	SR612	5.324	5.424	100.37	10.91	87.08	707.86	0.94	131.63
	6	158	IR15	41.567	41.667	118.14	24.19	84.46	483.08	0.78	120.18
	7	153	Decatur Blvd.	4.624	4.64	106.95	8.99	77.99	483.52	0.84	117.89
	8	158	IR15	41.667	41.767	105.53	22.24	74.20	409.34	0.69	105.58
	9	153	SR596	5.293	5.393	82.73	7.85	72.04	371.99	0.78	108.89
	10	153	Maryland Pkwy.	9.794	9.894	82.69	11.22	69.51	745.94	0.75	105.07

^*∗*^Crashes/mi/year; ^*∗∗*^crashes/mi^2^/year.
